# Supplementary material for: Determining the depth of surface charging layer of single Prussian blue nanoparticles with pseudocapacitive behaviors
Source: Nat Commun. 2022 Apr 28;13:2316. doi: 10.1038/s41467-022-30058-4 (PMC9051208; doi:10.1038/s41467-022-30058-4)
Supplement: Supplementary file 1 — Supplementary information [file 41467_2022_30058_MOESM1_ESM.pdf]

Supplementary Information

Determining the depth of surface charging layer of single Prussian blue nanoparticles with pseudocapacitive behaviors

Ben Niu[1], Wenxuan Jiang[1], Bo Jiang[1], Mengqi Lv[1], Sa Wang[1], Wei Wang[1]*

[1]State Key Laboratory of Analytical Chemistry for Life Science, Chemistry and Biomedicine Innovation Center (ChemBIC), School of Chemistry and Chemical Engineering, Nanjing University, Nanjing 210023, China. *Correspondence e-mail: wei.wang@niu.edu.cn

Table of Contents

1. Synthesis and characterizations of Prussian blue nanoparticles

2. Electrochemical system and optical configurations

3. The distribution of formal potential of PBNPs

4. The long-time cyclability of PBNPs during electrochemical modulation

5. The distinction between Faradaic component and non-Faradaic component

6. The calculation of optical amplitude and phase

7. The optical transfer function (OTF) defined by oEIS

8. The linear dependence of optical intensity with ion content

9. Extracting the depth of surface charging layer

10. The influence of the state-of-charge on oEIS

11. The influence of the electrical contact on oEIS

12. The equivalent circuit model of single PBNPs

13. Scattering intensity and equivalent radius of different PBNPs

14. The calculation of optical coefficient a

15. The equivalent circuit model of the entire ITO electrode

1. Synthesis and characterizations of Prussian blue nanoparticles

The Prussian blue nanoparticles (PBNPs) were synthesized according to a reported ultrasonic method[1]. 422 mg $K_4[Fe(CN)_6]\cdot3H_2O$ (1 mmol) powder was added into 0.1 mol/L hydrochloric acid aqueous solution. After immerging into water bath at 37 °C for 85 minutes under ultrasonic condition, the solution was cooled down to ambient temperature. The blue-green product was centrifuged at 14800 rpm to collect the deposit. Finally, the obtained colloidal was washed with deionized water (DIW, 18 MΩ·cm, Milli-Q, Thermo Fisher) and dried in the vacuum oven at 30°C for 12 hours.

The UV-vis extinction spectrum of PBNPs aqueous solution was measured by a Cary series UV-vis spectrophotometer (Agilent Technologies). The morphology was characterized by the scanning electron microscope (S-4800, Hitachi) and transmission electron microscope (JEM-2800, JEOL). The elemental analysis was done by X-ray photoelectron spectroscopy (PHI5000 VersaProbe).

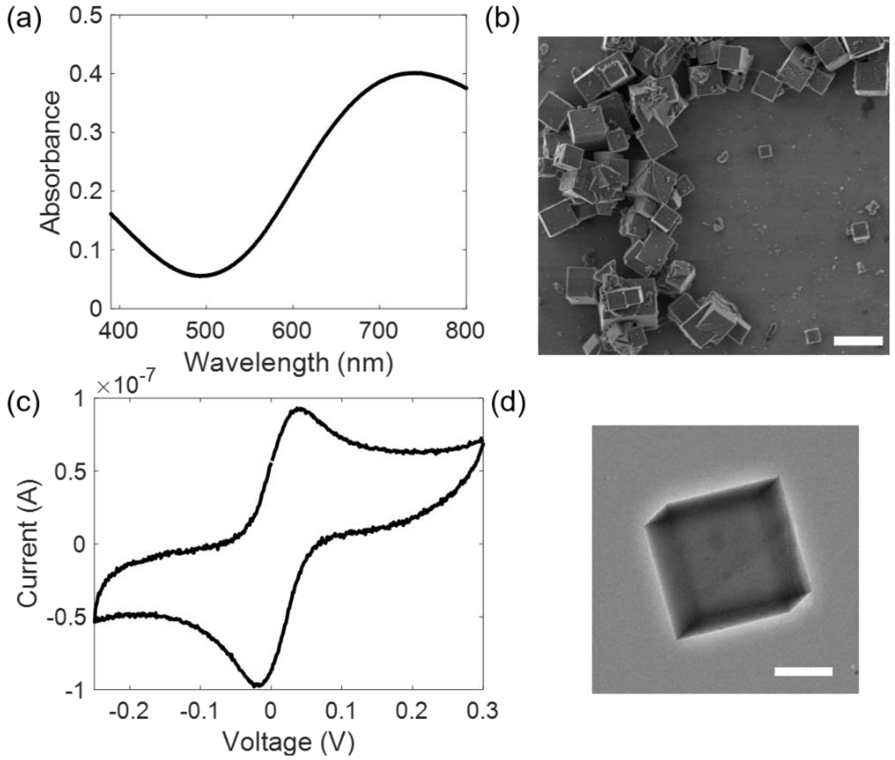

Supplementary Fig. 1 (a) UV-Vis spectrum of PB colloidal with a wide absorption band around 700 nm. (b) The SEM image of as-prepared PBNPs. Scale bar: 1 μm. (c) The cyclic voltammetry of PBNPs-deposited ITO electrodes. (d) The TEM image of PBNPs. Scale bar: 200 nm.

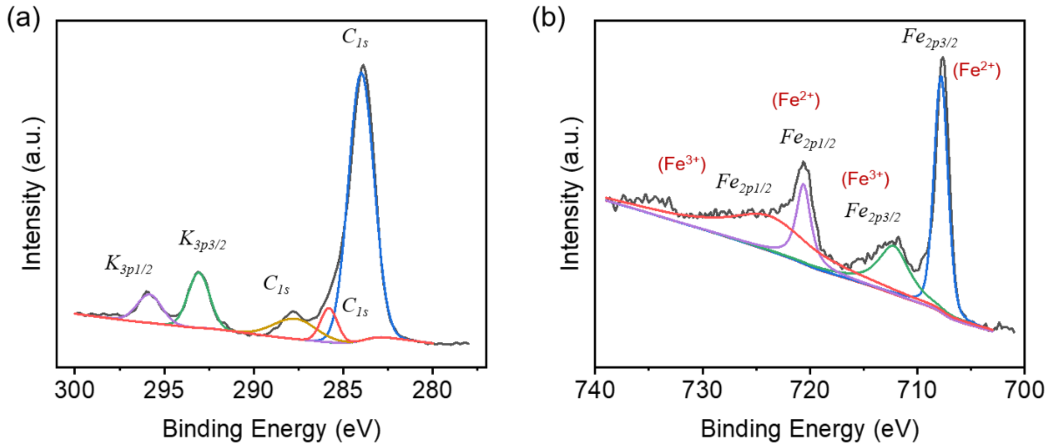

Supplementary Fig. 2 X-ray photoelectron spectra of synthesized PBNPs; (a) K_{3p} signals ($K_{3p1/2}$: 295.8 eV, $K_{3p3/2}$: 293.1 eV), C_{1s} signals (287.8 eV, 285.8 eV, 284.0 eV), and (b) Fe_{2p} signals ($Fe_{2p1/2}$: 725.0 eV, 720.6 eV, $Fe_{2p3/2}$: 712.2 eV, 707.8 eV).

2. Electrochemical system and optical configurations

The preparation of the electrochemical cell and electrodes were described in our previous work[2-3]. The thickness of ITO-coated glass slide was 1.1 mm (8 ohms/square, Wuhan Jinge-Solar Energy Technology Co. Ltd.). 150 μL droplet of 5 times diluted PBNPs solution was dropped on the ITO and dried in the vacuum oven (12 hours). The electrochemical measurements were performed in a 0.5 M KNO_3 solution in the absence of additional redox molecules. KNO_3 not only served as electrolyte to reduce IR drop, but also provided sufficiently high concentration of K^+ for insertion/extraction.

Voltage was applied by the potentiostat (Autolab PGSTAT302N, Metrohm AG) and modulated via an external waveform function generator (RIGOL, DG1000Z). A data acquisition card (USB-6281, National Instruments) was utilized to synchronize the voltage from potentiostat and transistor-transistor logic signals from the camera.

The optical image of PBNPs was obtained with an inverted microscope (Eclipse Ti-U, Nikon), which was installed with an oil-immersed dark-field condenser (NA = 1.20–1.43), an objective lens (40x, NA = 0.6), and a 660 ± 20 nm light-emitting diode (M660L3-C1, Thorlabs) as the light source (Supplementary Fig. 3a). The dark-field image was collected by a CCD camera (Stingray, Allied Vision Technologies). In this condition, we can visualize single PBNPs as small as 100 nm with sufficient contrast in DFM (Supplementary Fig. 4).

A grating spectrometer (Acton Spectra Pro SP-2300, Princeton Instruments) with slit was used to capture the scattering spectra. Supplementary Fig. 3b shows the scattering spectra of PB and PW states at different voltage, with maximum scattering wavelength near 700 nm. It was believed that the scattering of PBNPs at ~700 nm was due to the Rayleigh resonant scattering, because the incident wavelength was consistent with the absorption band of Fe-Fe intervalence charge transfer[3]. When applying negative voltage, the PBNPs can be transformed into PWNPs with an obvious decrease in the scattering intensity around 700 nm.

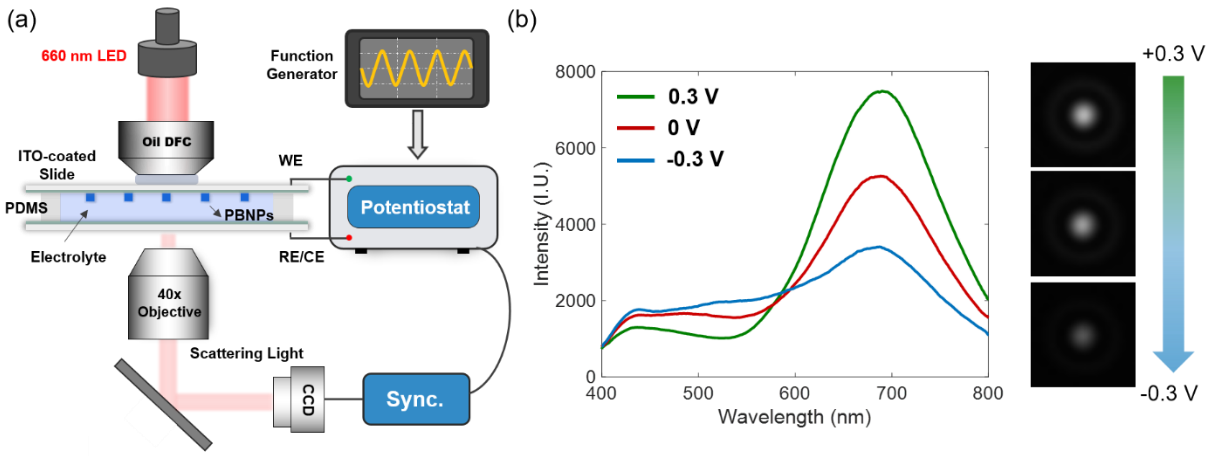

Supplementary Fig. 3 (a) Detailed schematic diagram of the optical-electrochemical combined device. (b) Dark-field scattering spectra of a single PBNP under different voltage conditions.

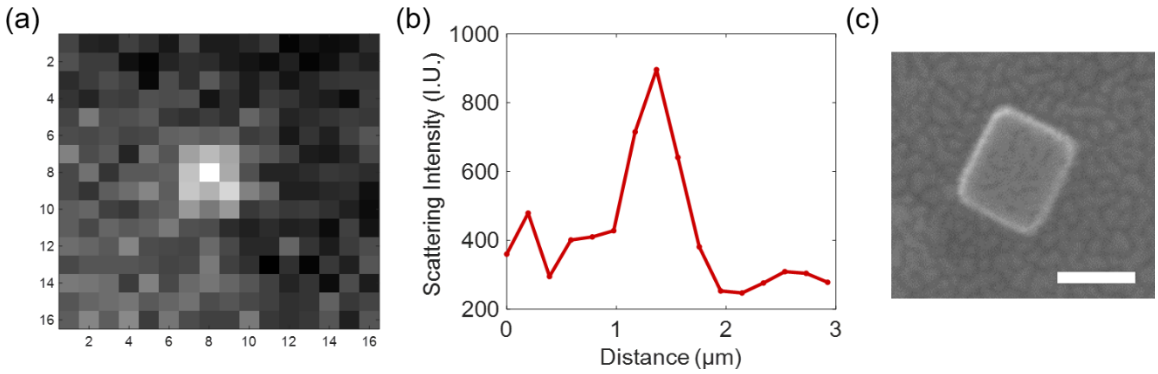

Supplementary Fig. 4 The smallest nanoparticle that can be visualized by our monochromatic dark-filed microscopy in the field of view. (a) Dark-field scattering image. (b) The corresponding intensity profile of the dark-field image. (c) The corresponding SEM image (scale bar: 100nm).

3. The distribution of formal potential of PBNPs

Here, we defined the formal potential of PBNPs for better understanding the stage-of-charge of PBNPs. The formal potential of each individual had been examined by monitoring the optical intensity as a function of potential. When recording a series of optical imaging under sweeping voltage (-300 mV – 250 mV with a sufficiently slow scan rate of 5 mV/s), the formal potential was determined at 50% between the highest and lowest intensity (Fig. 1c). It was a consequence of structural and compositional heterogeneity during synthesis. We have accordingly selected the ones with formal potential in the range between -0.05 and 0 V for further study, which accounted for the largest portion (Supplementary Fig. 5). The subsequent frequency modulation experiment was conducted at the offset potential of -25 mV for the largest optical amplitude.

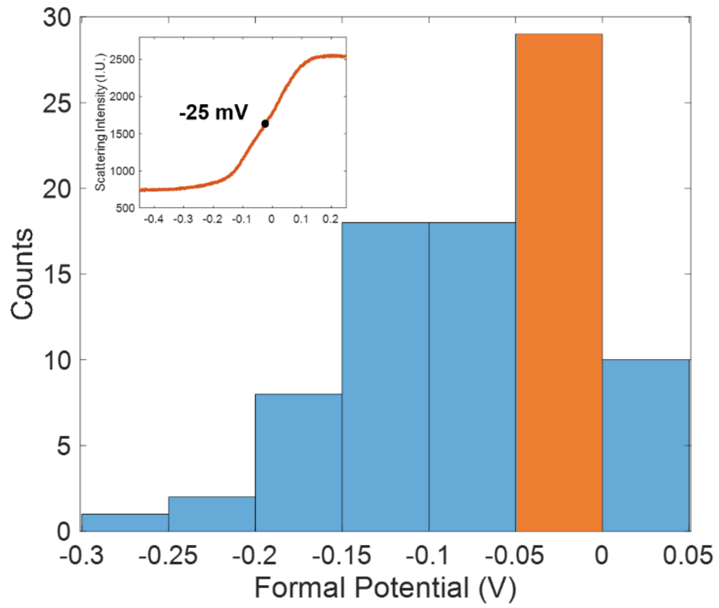

Supplementary Fig. 5 The distribution of formal potential of around 100 PBNPs, the inset is the representative formal potential of -25 mV.

4. The long-time cyclability of PBNPs during electrochemical modulation

In order to demonstrate the stability of PBNPs during the long-time experiment, PBNPs were modulated during 1000 consecutive cycles at a frequency of 1 Hz (17 minutes). The Fourier transform was utilized to extract the optical amplitude (detailed process were shown below). We exemplified the Fourier transform results by calculating the 400-500th cycles (the green box in Fig. 1f).

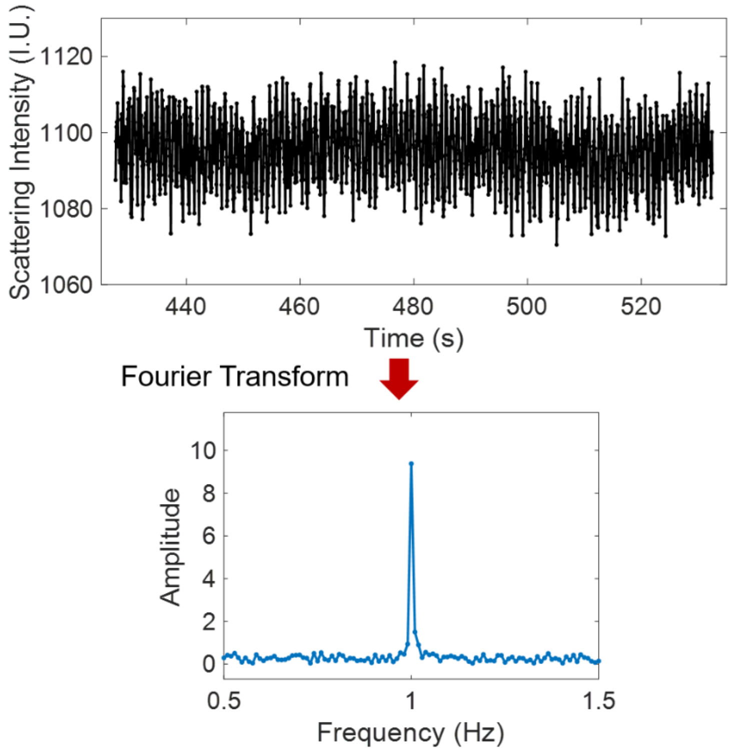

Supplementary Fig. 6 The fluctuation of scattering intensity during 100 consecutive cycles (400-500th cycles) at a frequency of 1 Hz, the diagram below is the amplitude after Fourier transform.

5. The distinction between Faradaic component and non-Faradaic component

The dark-field imaging method can effectively distinguish Faradaic and non-Faradaic components. When a sinusoidal voltage was applied, the scattering intensity of particles could change with the voltage, while the intensity of the ITO electrode remained unchanged (Supplementary Fig. 7). Therefore, the redox reaction of PBNPs and charging/discharging process of electric double layer can easily be distinguished, only by selecting the rectangle region of interest (ROI) at the position of single PBNPs (red rectangle, left panel in Supplementary Fig. 7).

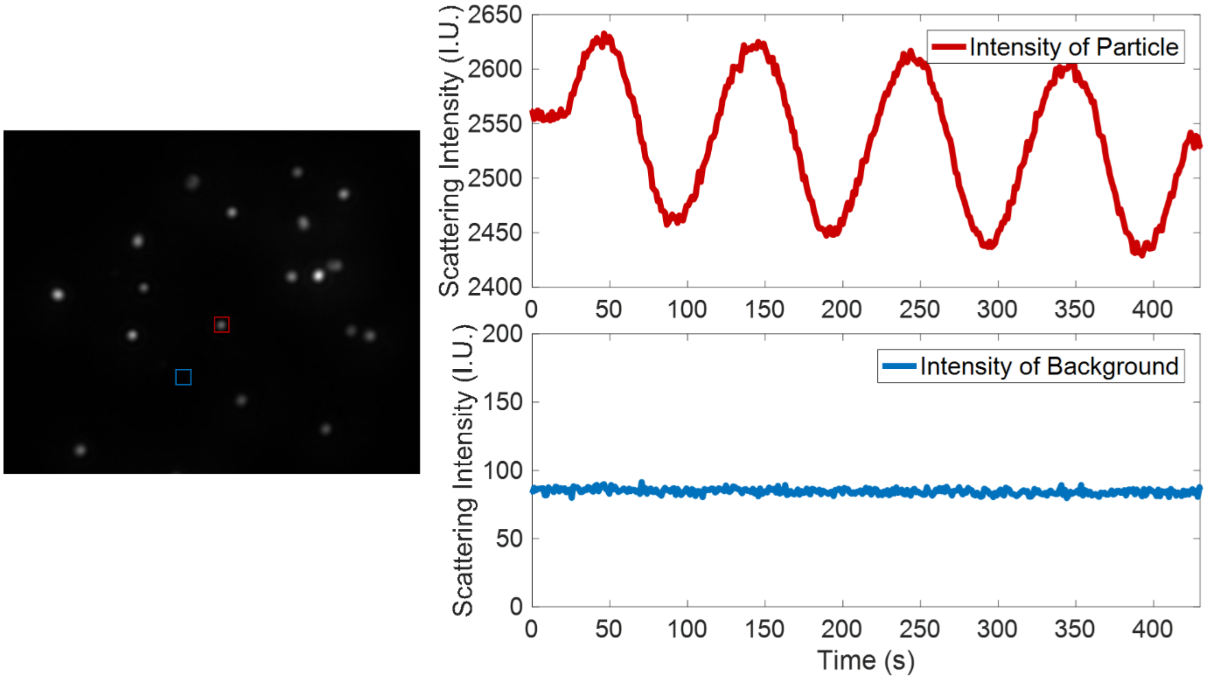

Supplementary Fig. 7 The scattering intensity of PBNPs and surrounding background when a sinusoidal voltage was applied. The red area represents a single PBNP, the blue area is the background of ITO electrode. The right panel is the corresponding scattering intensity. The modulation frequency was 0.01 Hz and the amplitude was 20 mV.

6. The calculation of optical amplitude and phase

We then extracted the amplitude and phase of optical intensity curves. In the specific operation, it should be noted that in order to ensure the linear response of system, a small voltage was necessary. However, the small voltage would also result in a low signal-to-noise ratio of optical amplitude. Under this limitation, we optimized the amplitude of voltage at each frequency to meet the sufficient signal-to-noise ratio. In the low-frequency region, the voltage was small, while in the high-frequency region, the voltage was required to be larger. Here, we had selected four representative frequencies, 0.1 Hz (Supplementary Fig. 8a), 1 Hz (Supplementary Fig. 8b), 10 Hz (Supplementary Fig. 8c) and 100 Hz (Supplementary Fig. 8d), to show the fluctuations of scattering intensity under different voltages.

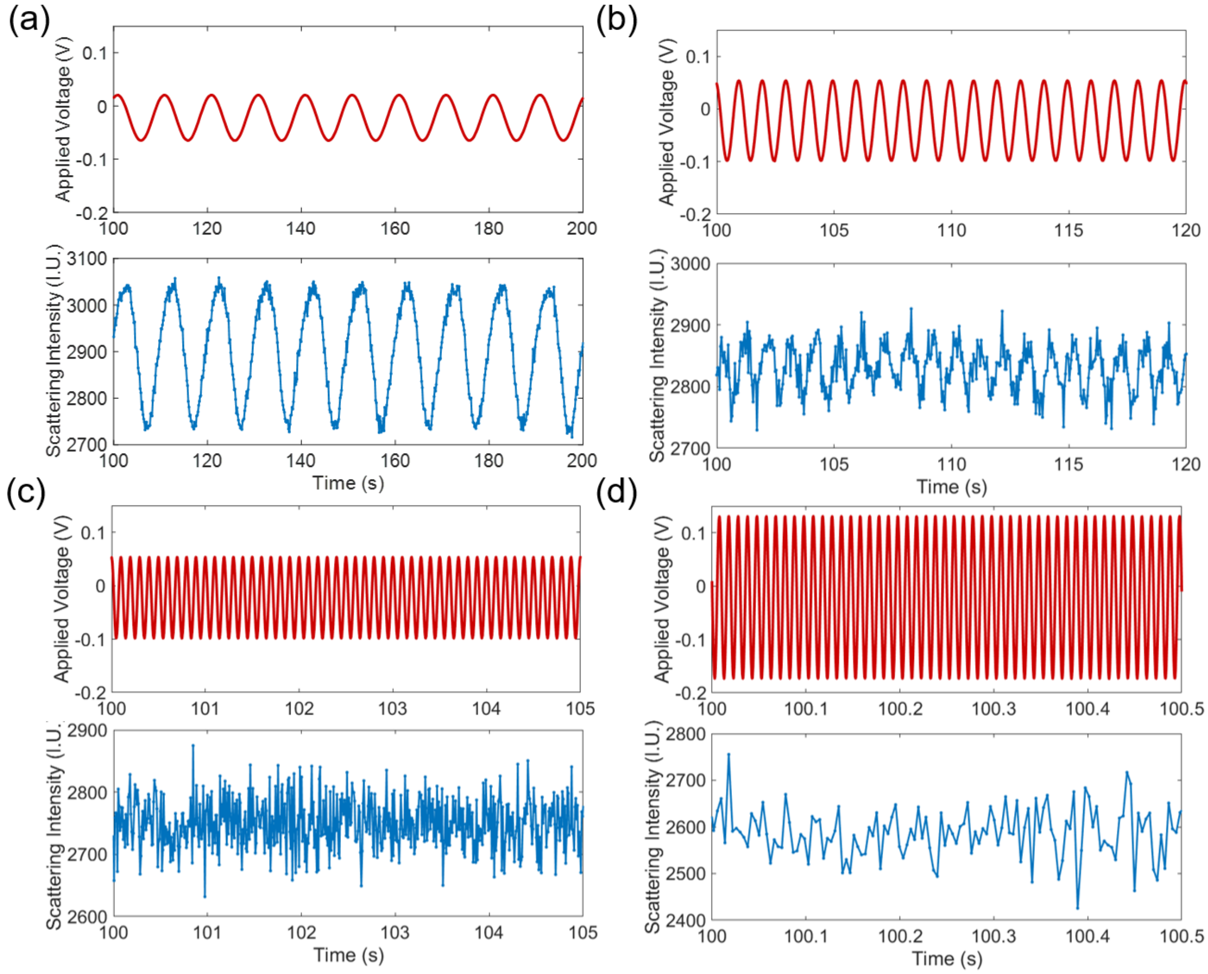

Supplementary Fig. 8 (a) Schematic diagram of the fluctuation of the scattering intensity when the modulation frequency is 0.1 Hz and the voltage is ±45 mV; (b) The modulation frequency is 1 Hz and the voltage is ±80 mV; (c) The modulation frequency is 10 Hz and the voltage is ±80 mV; (d) The modulation frequency is 100 Hz and the voltage is ±160 mV.

The Fourier transform was utilized to extract the optical amplitude and phase. Whether it was at low frequency (0.1 Hz) or high frequency (100 Hz), sufficient signal-to-noise could be obtained.

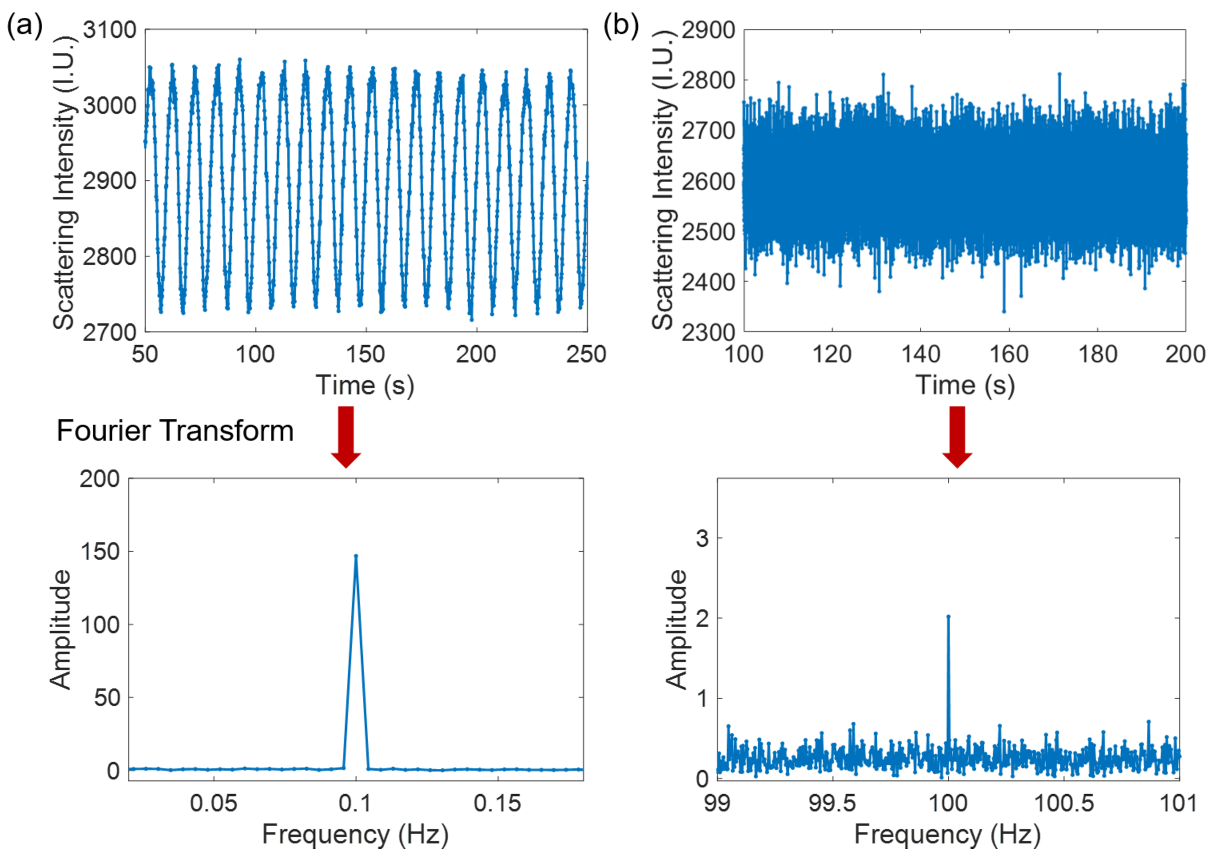

Supplementary Fig. 9 The optical amplitude calculated by Fourier transform when the modulation frequency is (a) 0.1 Hz; (b) 100 Hz.

7. The optical transfer function (OTF) defined by oEIS

The optical transfer function (OTF) that we measured in Fig. 2a and Fig. 2b was defined as following:

$$Optical\,Transfer\,Function\,(OTF) = \frac{\Delta I}{V}.$$

Here, ΔI is the optical amplitude and V is the voltage. In the subsequent processing, we had unified the amplitude of the voltage at each frequency. In other words, all the optical amplitude was calculated at the amplitude of 20 mV of voltage (Fig. 2a). The optical phase difference was the optical phase minus voltage phase (Fig. 2b).

We chose to display the OTF rather than impedance directly because of the following reasons. In order to obtain the impedance/admittance information, a first-order derivative had to be performed to the optical intensity curves (corresponding to charge quantity) to resolve the current (corresponding to charge transfer rate). Unfortunately, the first-order derivative was found to significantly increase the noise level, particularly at high-frequency range (Supplementary Fig. 10b). It made the quantification at high frequency range challenging because the signal itself became smaller when the frequency was higher.

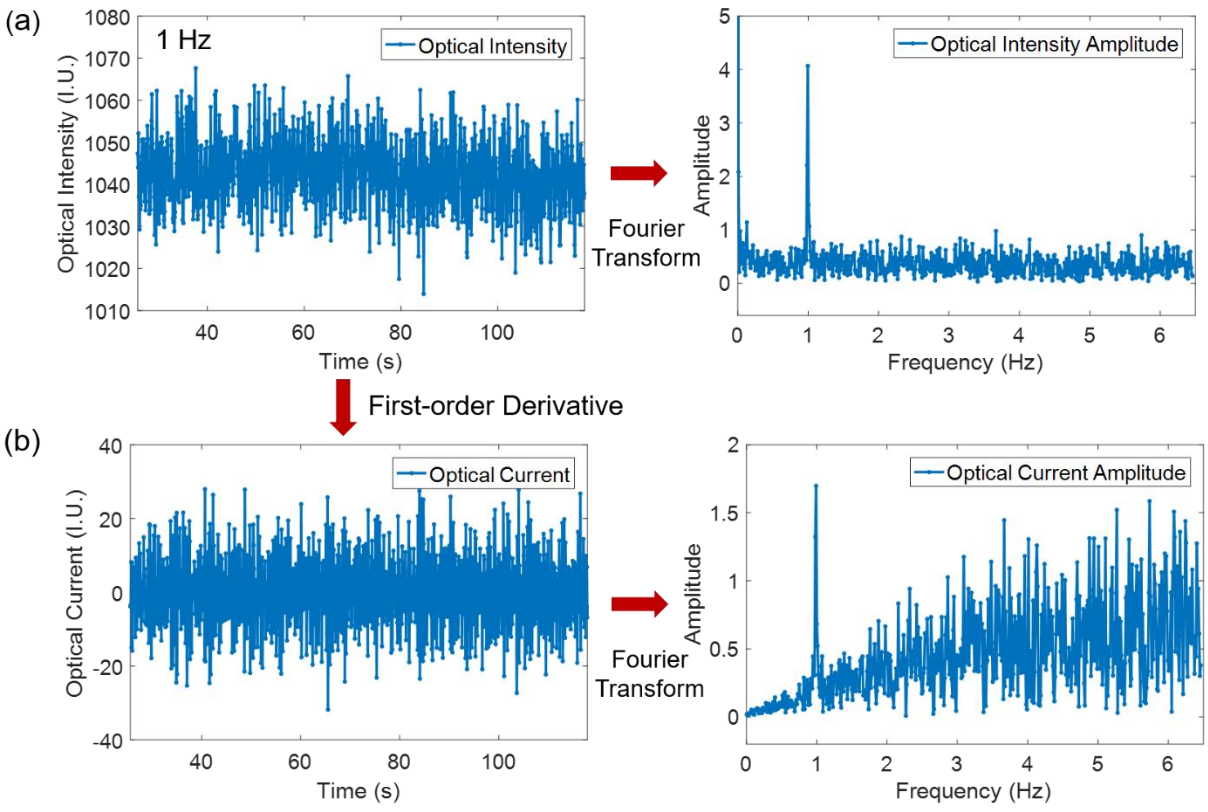

Supplementary Fig. 10 Left panel: (a) Representative scattering intensity curve under a modulation frequency of 1 Hz and (b) its first order derivative. The corresponding Fourier transform results are shown in the right panel.

Although it was difficult to directly measure the optical current at each frequency, we could also build a mathematic transform to obtain the impedance/admittance (based on voltage ~ current) from OTF (charge quantity or the integration of current ~ voltage).

The amplitude and phase of the impedance can be written as:

$$|Z| = \frac{V}{I} = \frac{V}{Q \cdot 2\pi f} = \frac{a}{2\pi f \cdot |OTF|} \propto \frac{1}{f \cdot |OTF|},$$

$$\Phi_Z = \Phi_V - \Phi_I = \Phi_V - \Phi_Q - \frac{\pi}{2} = -\Phi_{OTF} - \frac{\pi}{2}.$$

The good fitted results in Supplementary Fig. 11 show the reliability of this conversion.

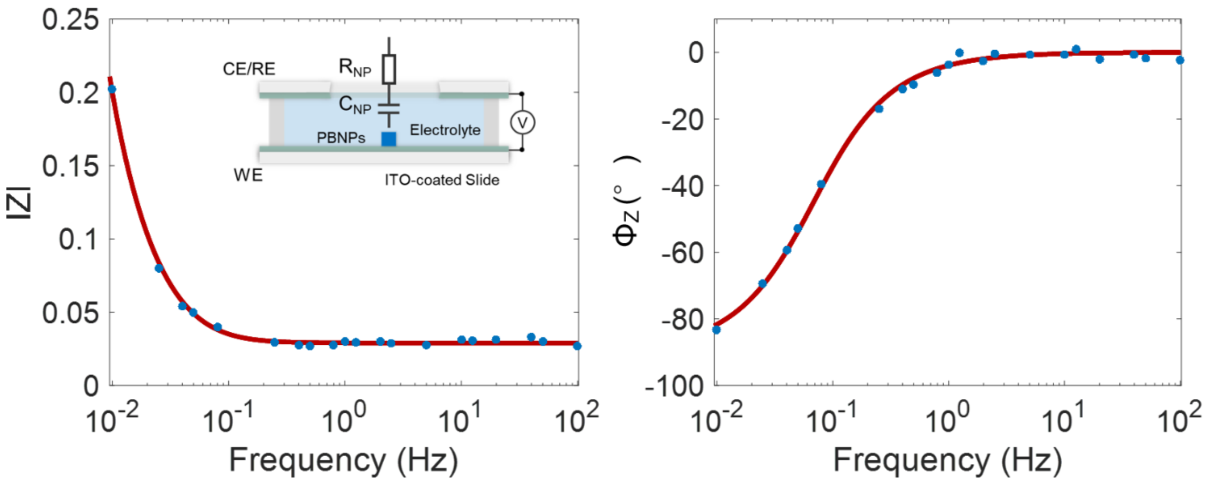

Supplementary Fig. 11 The amplitude (|Z|, left panel) and phase (Φ_Z, right panel) of impedance can be calculated from those of OTF. The red lines are fitted results and the blue dots are experimental data.

Furthermore, we used the Kramers-Kronig methods to verify the reliability of the frequency-dependent data:

$$\Phi_{OTF}(\omega) = -\frac{\omega}{\pi}\int_0^{\infty}\frac{In(|OTF(x)|)}{x^2-\omega^2}dx,\ \ In(|OTF(x)|) = \frac{2}{\pi}\int_{-\infty}^{\infty}\frac{\varphi_{OTF}(\omega)}{x-\omega}dx.$$

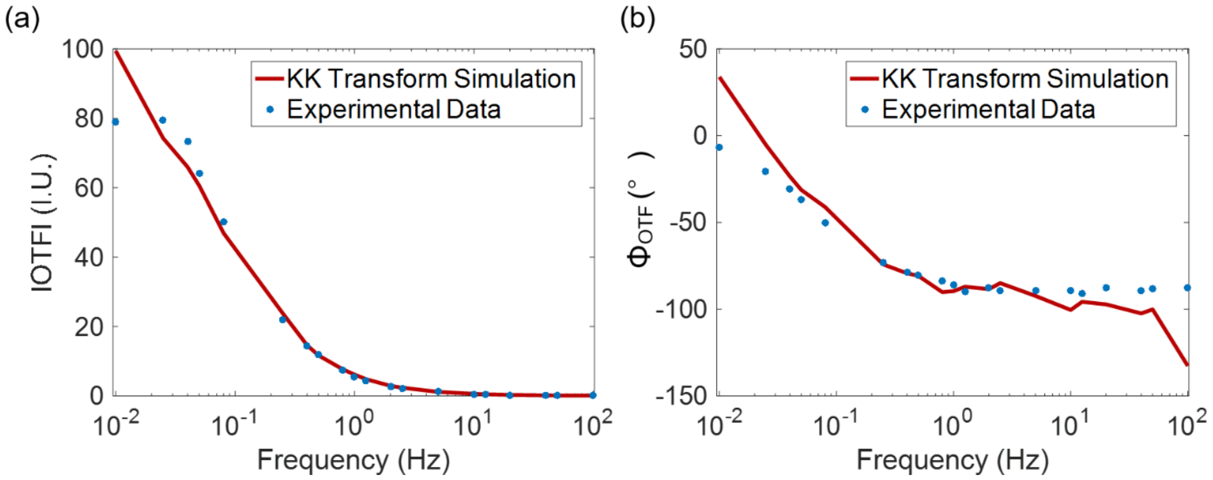

Supplementary Fig. 12 Bode plots of OTF amplitude (a) and phase (b). The red lines are Kramers-Kronig transform simulation results, and the blue dots are experimental data.

8. The linear dependence of optical intensity with ion content

In order to convincingly demonstrate that the overall scattering signal of single PBNPs (smaller than optical diffraction limit) was quantitatively dependent on its state-of-charge, we have performed the experiment by simultaneously recording the faradaic current and optical traces of one PBNP during its collision. The apparatus and methodology were adopted from our previous publication[4]. While $LiCoO_2$ nanoparticles were studied in previous study, herein PBNPs was used instead. Briefly, a 50×50 μm^2 microelectrode was fabricated to reduce the background current. When applying a constant reduction potential (-300 mV) onto the electrode and allowing single freely-moving PBNPs in the suspension to stochastically collide onto the electrode, a transient reduction current was recorded after the collision-and-stay of single PBNPs. By doing so, it was ensured that the electrode current was solely contributed by the particular nanoparticle. Since early 2000s, it has been a very powerful strategy pioneered by Lemay, Bard, Compton, and many others[5], which is known as single nanoparticle collision/impact electrochemistry. Our contribution was to employ an optical microscopy to simultaneously record the entire collision-and-reaction process, and to quantitatively compare the optical signals with electrochemical current.

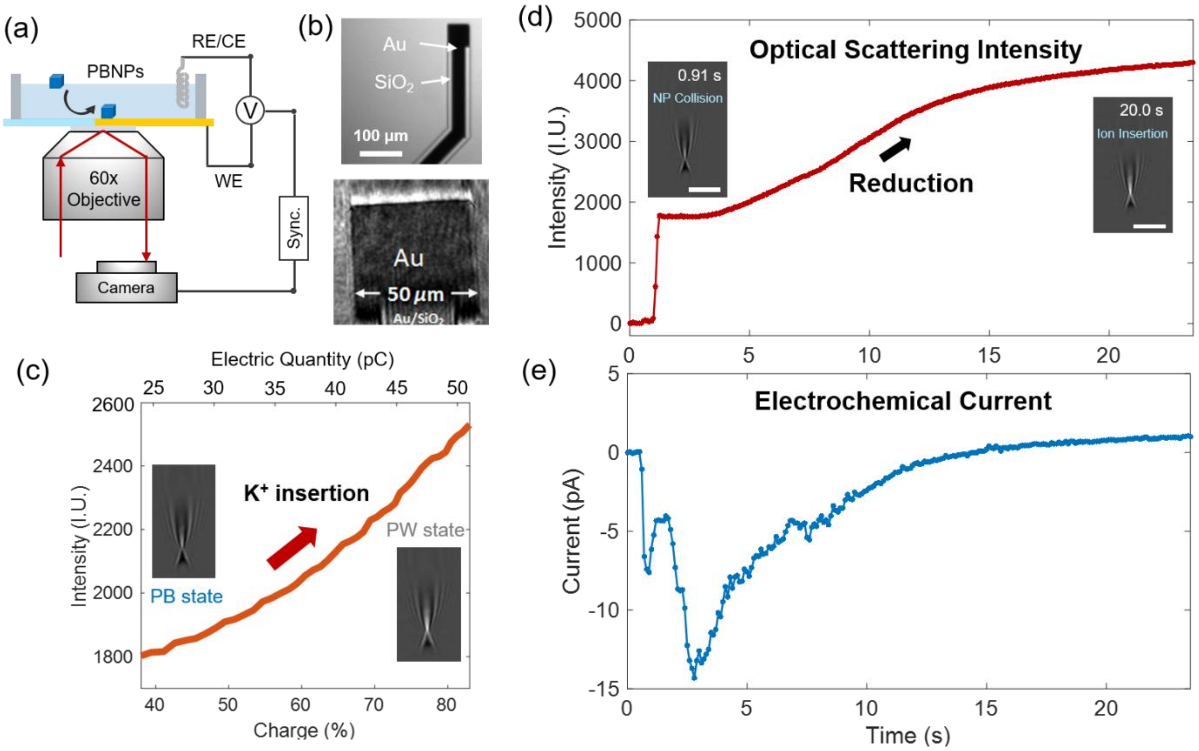

Supplementary Fig. 13 (a) Schematic illustration of single PBNP collision events with correlated surface plasmon resonance microscopy (SPRM) imaging and electrochemical recording. The SPRM was equipped with a high numerical aperture oil immersion objective and a red p-polarized light. (b) The bright-field (top panel) and plasmonic images (bottom panel) of Au microelectrode. (c) Correlation between the state-of-charge/electric quantity and the SPRM intensity for the PBNP. Transient SPRM intensity curve (d) and sequential electrochemical current (e) of a single PBNP, scale bar: 5 μm.

As shown in Supplementary Fig. 13d, at 0.91 second, the nanoparticle collided on the electrode and therefore led to a sudden increase in the optical signal. Then, electrochemical reduction of the nanoparticle gradually increased the optical signal, indicating the gradual conversion from PB to PW. This point was confirmed by the simultaneously recorded reduction current (Supplementary Fig. 13e). In this experiment, it was ensured that the optical signal and electrode current was from the same individual PBNP[4]. If we plotted the optical intensity as a function of quantity of injected electrons (integration of current), there was a quasi-linear dependence of optical scattering signal on the state-of-charge, especially in the range between 40−80% (Supplementary Fig. 13c).

It was necessary to clarify that, surface plasmon resonance microscopy (SPRM) rather than dark-field microscopy (DFM) was employed to obtain the results shown in Supplementary Fig. 13a. It was because a micron-sized electrode was required for this experiment to suppress the background current. The edge of microelectrode resulted in a rather high optical background in DFM. However, since both SPRM and DFM measured the optical scattering signal, we believed the monotonic dependence of optical scattering on state-of-charge remained valid in both cases.

When charging single nanoparticles, we would like to believe that the overall scattering intensity was able to quantitatively report the state-of-charge of single nanoparticle as long as it was smaller than the optical diffraction limit (~300 nm), although with the existence of the spatiotemporal heterogeneity of the electrode. First, our results in single nanoparticle collision electrochemistry had clearly supported this point for PBNPs. Second, nearly all individual PBNPs we investigated displayed a monotonic and smooth intensity curve during

electrochemical charging/discharging cycles. In addition, in our previous study on single $LiCoO_2$ nanoparticles (~200 nm size), monotonic dependence was also observed[4].

There were a few reasons to ensure a more straightforward and quantitative relationship between the optical signal and the ion insertion content in our study. First, because as-prepared PBNPs were around 100~300 nm and of regular cubic-shape, the diffraction effect allowed for accessing the overall change in morphology and refractive index. This scenario was in contrast to micro-particles with a size of tens of microns and irregular morphology[6], which was superior to map the ion transport pathways with sub-particle spatial resolution by imaging the local variations of scattering from an irregular 10-micron sized $LiCoO_2$ particle. Second, it was believed that the scattering of PBNPs at ~700 nm (wavelength) was due to the resonant Rayleigh scattering, because the incident wavelength was consistent with the absorption band of Fe-Fe intervalence charge transfer. Since absorption of nano-sized object was less sensitive to its morphology, it would be more reliable to reflect the overall ion content within the entire nanoparticle.

In summary, the linear dependence between optical scattering intensity of single PBNPs and its state-of-charge was reliable under these two conditions: 1) nanoparticle size was smaller than optical diffraction limit, and 2) the state-of-charge was close to 50% in our experiment.

9. Extracting the depth of surface charging layer

In order to further prove that the $\Delta I \propto f^{-1}$ in high frequency region and $\Delta I \propto f^{-0.5}$ in low frequency region, we compared the double logarithm graph. The slope of 1 indicated that the optical amplitude conformed to the following formula:

$$\Delta I = k_1 \cdot \frac{1}{f}, \;\; lg(\Delta I) = lgk_1 + lg\,(1/f).$$

The slope of 0.5 indicated that the optical amplitude conformed to the formula:

$$\Delta I = k_2 \cdot (\frac{1}{f})^{0.5} + b, \; lg(\Delta I - b) = lgk_2 + 0.5 \times lg(1/f).$$

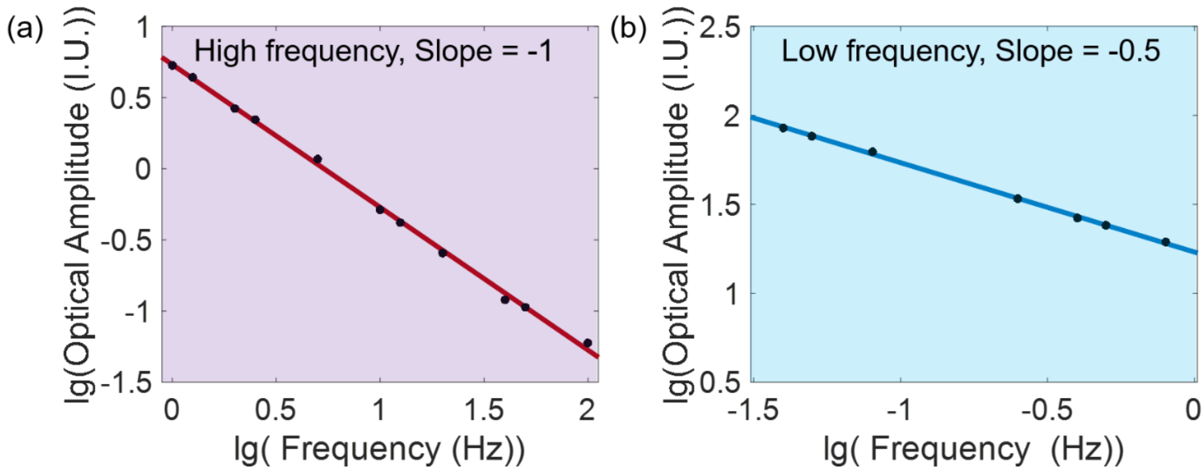

Supplementary Fig. 14 (a) The dual-logarithm diagram confirming that the $\Delta I \propto f^{-1}$ in high-frequency region (a) and $\Delta I \propto f^{-0.5}$ in low-frequency region (b). The lines are the fitted results, and the black dots are experimental data.

Once plotting the optical amplitude as a function of the inverse of the square root of the frequency ($f^{-0.5}$), it became clear that the curve was composed of two segments: a linear curve in the low frequency range (right part, $f^{-0.5}$), and a parabolic curve in the high frequency range (left part, f^{-1} or $(f^{-0.5})^2$). It was well consistent with the proposed mechanism. In order to unbiasedly determine the corner frequency, a piecewise function (in which f_{cutoff} is a parameter-to-be-fitted) was applied to fit the entire curve:

$$|OTF|\,(f) = \begin{cases} k_1 f^{-1} & for \quad f \geq f_{cutoff} \\ \\ k_2 f^{-0.5} + b & for \quad f \leq f_{cutoff} \end{cases}$$

$$f = f_{cutoff} \qquad k_1 f_{cutoff}{}^{-1} = b + k_2 f_{cutoff}{}^{-0.5} \quad .$$

For the representative amplitude results shown in Fig. 2a, the corner frequency was fitted to be 0.9 Hz.

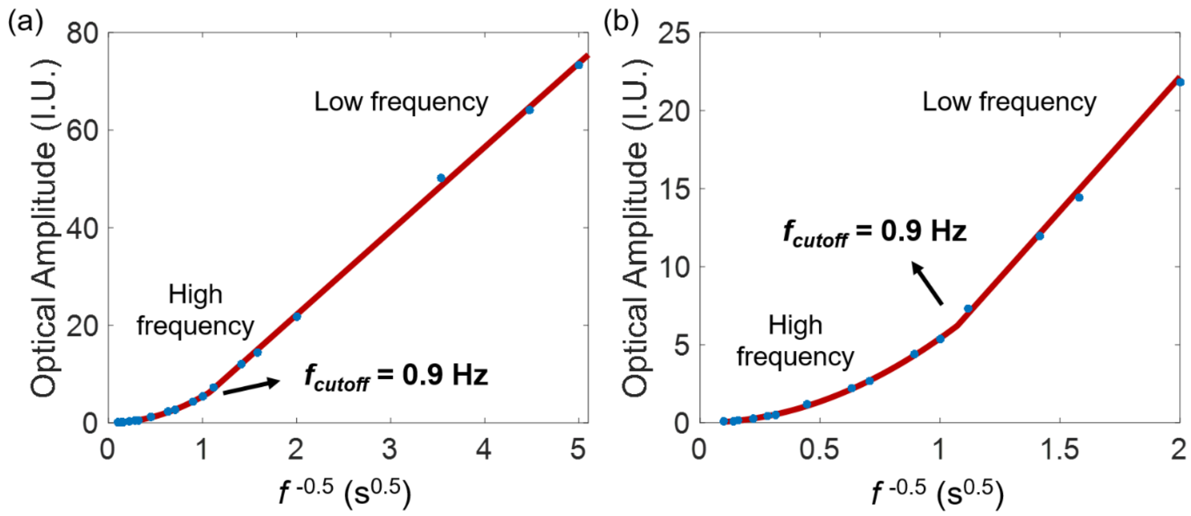

Supplementary Fig. 15 (a) When plotting optical amplitude as a function of $f^{-0.5}$, the dependence can be well fitted by a piecewise function above. (b) A part of (a) is enlarged to better display the transition between two trends at f_{cutoff} of 0.9 Hz. The red lines are the fitted results, and the blue dots are experimental data.

So the contribution from surface charging (Q_s) is calculated to be 5.9 IU. Since the total charge capacity (Q_t) is 82.2 IU, the contribution percentage of pseudocapacitive behavior is 7.2%.

The single PBNP has regular cuboid geometry that is well characterized by its length (215 nm), width (200 nm) and height (215 nm). Since that PBNP exhibits a face-centered cubic structure and its crystalline size is 1.02 nm, the total number of cells in PBNP are $(\frac{215}{1.02}) \times (\frac{215}{1.02}) \times (\frac{200}{1.02})$. Therefore, the number of cells at near-surface region must reach $2.4 \times \left(\left(\frac{215}{1.02} \times \frac{215}{1.02}\right) \times 4 + \left(\frac{200}{1.02} \times \frac{215}{1.02}\right) \times 2\right)$. That is to say, the depth of surface charging layer is around 2.4 unit cells. Note that the actual depth can be smaller than 2.4 unit cells when considering the surface roughness of PBNPs.

The corresponding SEM and AFM images of six PBNPs mentioned in Figure 3a-f were shown in Supplementary Fig.16. The six individual was selected to cover different sizes ranging from 100 to 300 nm, and their height and length were on the same level. They all exhibited capacitor-like behavior in high-frequency range and battery-like behavior in low-frequency range.

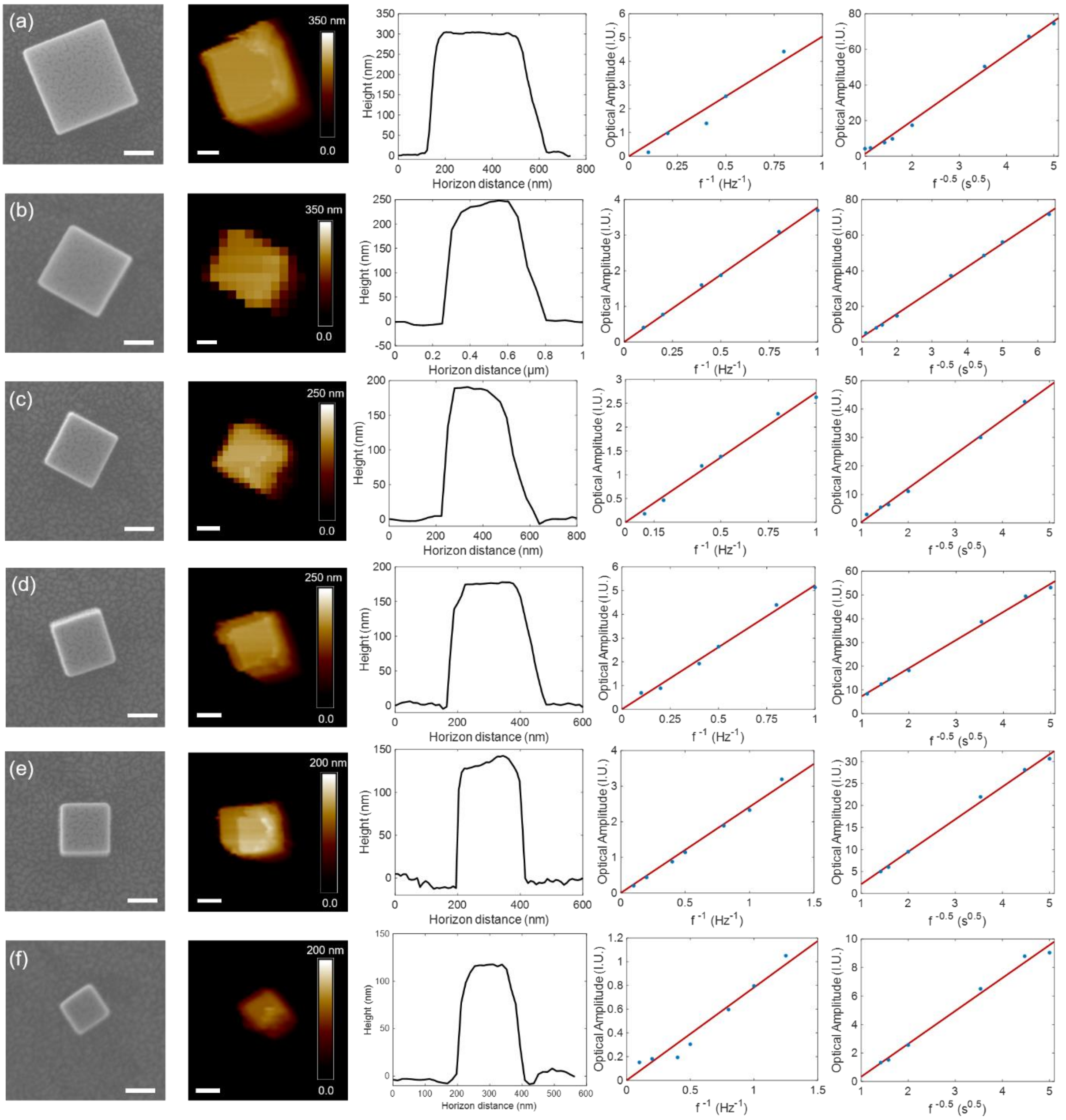

Supplementary Fig. 16 (a) – (f) The geometry and fitting results of six PBNPs. From left to right: SEM image (scale bar: 100 nm), AFM image (scale bar: 100 nm), the corresponding height profile, fitting results of optical intensity in high-frequency region and low-frequency region, respectively. The red line shows the fitted results, and the blue dots are experimental data.

10. The influence of the state-of-charge on oEIS

The pseudo-capacitive behavior was significantly dependent on the state-of-charge. According to this, we had conducted further experiments at different offset potentials to examine the influence of state-of-charge on the OTF as well as corner frequency (f_{cutoff}).

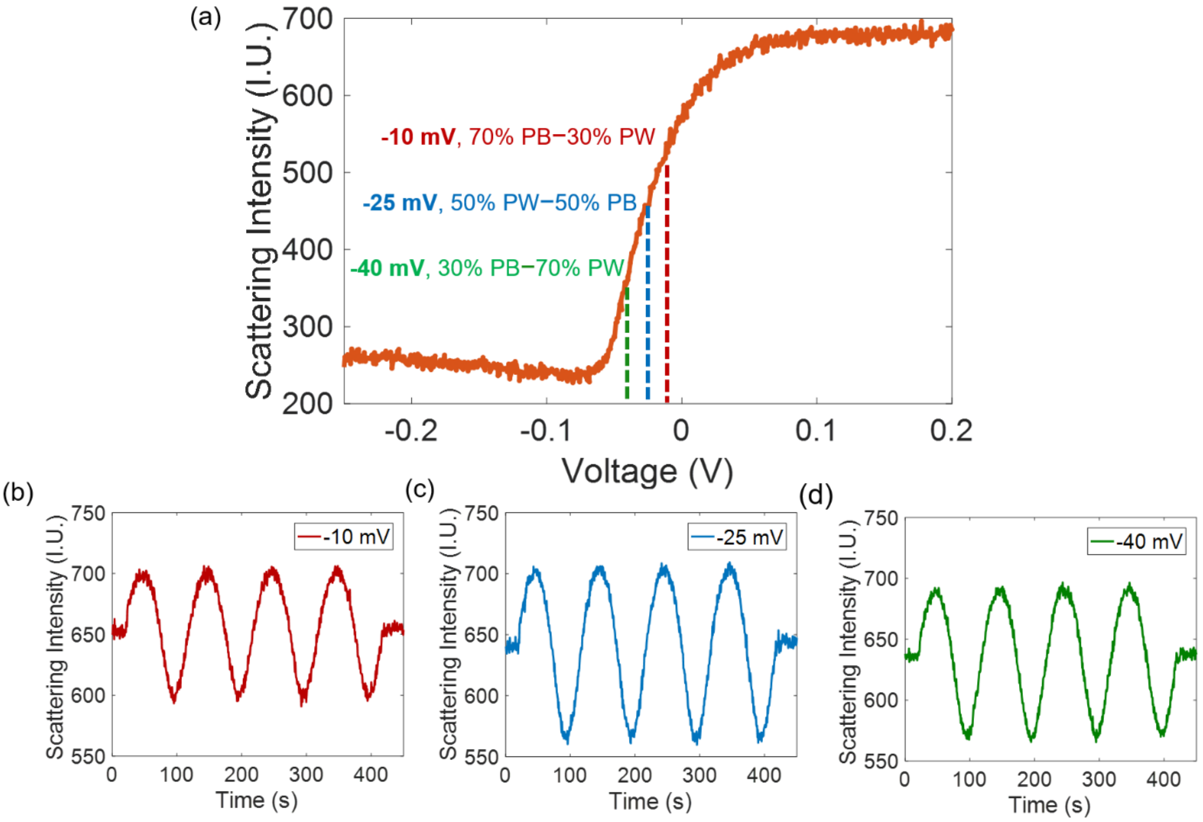

Supplementary Fig. 17 (a) The dependence of the scattering intensity of single PBNPs on the potential. Scan rate is 5 mV/s. Representative scattering intensity curves are provided when the modulation frequency is 0.01 Hz with the same amplitude of 20 mV, and corresponding offset potentials are -10 mV (b), -25 mV (c), and -40 mV (d), respectively.

First, optical response as a function of sweeping potential from -300 to +250 mV (scan rate: 5 mV/s) was shown in Supplementary Fig. 17a. It revealed a formal potential of -25 mV, and a quasi-linear dependence of optical intensity with potential between -55 and 5 mV.

Then, oEIS of the same individual PBNP was measured at varying offset potentials of -10, -25, -40 mV with the same amplitude of 20 mV. The results were shown in Supplementary Fig. 18. According to the curve above, the state-of-charge under these potentials was 70% PB−30% PW, 50% PB−50% PW, 30% PB−70% PW, respectively. It was clear that the maximal optical amplitude was obtained at the formal potential of -25 mV.

Similar corner frequency was also observed in the state-of-charge range of 30%−70%, 0.7 Hz (-10 mV), 0.8 Hz (-25 mV) and 0.8 Hz (-40 mV), contributed 7.1%, 7.6% and 7.4% to the total charge storage capacity, respectively. In other words, the corner frequency (~0.8 Hz) and the depth of surface charging layer (~2 unit cells) were more or less independent on the state-of-charge, at least in the range of formal potential ± 15 mV (corresponding to state-of-charge 30~70%). We attributed such stability as the same face-centered cubic crystal structure and the similar lattice parameters between Prussian Blue (oxidized form) and Prussian White (reduced form).

At the same time, the difference in corner frequency should be more evident when an extreme state-of-charge (such as 10% and 90%) was examined, or in another redox system with significant lattice change during cycling. Unfortunately, when we tried to apply such extreme conditions in our study, the sample tended to rapidly lose activity (fading) during consecutive and long cycling under extreme potentials.

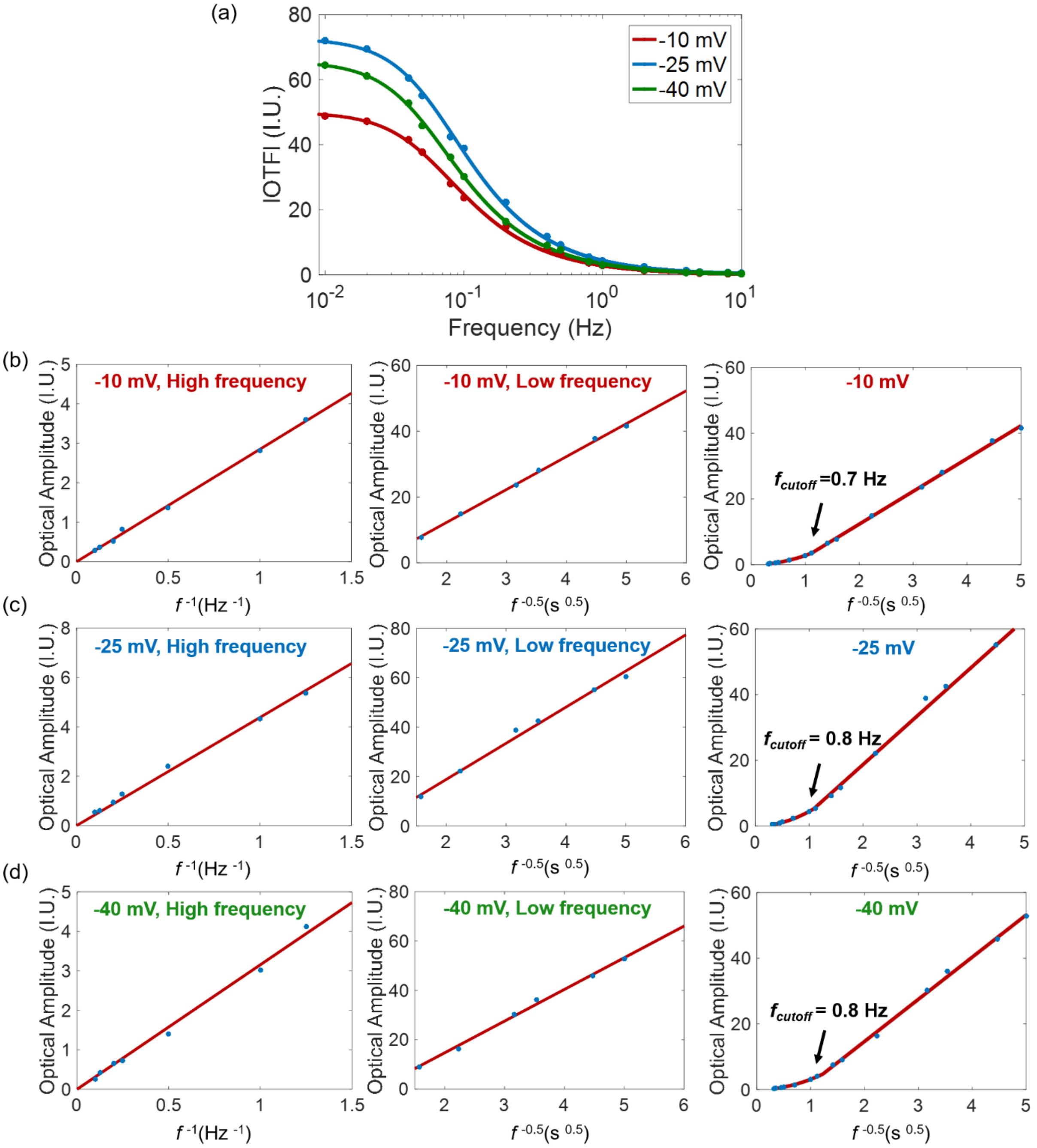

Supplementary Fig. 18 (a) The OTF amplitude of the same individual PBNP at varying offset potentials of -10 mV, -25 mV and -40 mV. Surface-limited behaviour in the high frequency region (left panel) and diffusion-limited behaviour in the low frequency region (middle panel) at -10 mV (b), -25 mV (c) and -40 mV (d) are shown respectively. The piecewise function fitting graphs are introduced to better display the corner frequency (right panel). The lines are fitted results and the dots are experimental data.

Moreover, the charge transfer resistance (R_{ct}) is related to the content of PB and PW component in one single nanoparticle:

$$R_{ct} = \frac{RT}{nFi_0}, \ while \ i_0 = FAk_0 C_R^{*\,\alpha} C_O^{*\,(1-\alpha)} \ .$$

Here, R is the gas constant, T is the temperature, n is the number of electron transferred, i_0 is the exchange current density, C_R is the concentration of the oxidation species, C_o is the concentration of the reduction

species, F is the Faraday constant, A is the surface area, k_0 is the standard electrochemical rate constant which is independent of the potential, and α is transfer coefficient which can also be regarded as constant approximately in a narrow potential window.

Finally, R_{ct} is considered to be concentration (or applied potential) dependent, which has the minimum value when the concentration of oxidation species and reduction species are equal. This result is in good agreement with our experimental results, the values of R_{NP} are 0.056 (-10 mV), 0.036 (-25 mV) and 0.047 (-40 mV), respectively.

11. The influence of the electrical contact on oEIS

The electrical contact is critical in the electrochemical experiment. Our previous work developed a sputter-coating method by depositing an ultrathin platinum layer[7], which investigated the influence of electrical contacts on the apparent activity of single nanoparticles. We clarify that this point remained valid, and the present oEIS offers a promising capability to quantify the contact resistance by analyzing R_{NP}.

As an example, we collected and compared the oEIS of a very same individual before and after drying the sample in a vacuum chamber (10^{-4} Pa) by 1 hr. As shown in Supplementary Fig. 19 below, the value of R_{NP} was found to ~20 times lower after the vacuum drying. This result not only demonstrated the capability of oEIS for quantifying the contact resistance of single nanoparticles, but also provided a more feasible protocol to enhance the electrical contacts by vacuum drying (than our previous method of metal sputtering).

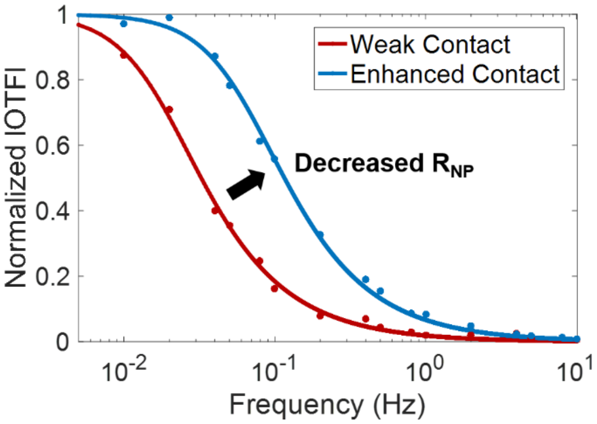

Supplementary Fig. 19 The normalized |OTF| of a very same individual PBNP before (red) and after (blue) vacuum drying. The lines are the fitted results, and the dots are experimental data.

In conclusion, nanoparticle capacitor (C_{NP}) shown in Fig. 4b described the charge storage capability of single PBNPs, including both the surface-limited charging layer and the diffusion-limited interior part. It was proportional to the volume of nanoparticles (Fig. 3g). Nanoparticle resistor (R_{NP}) was used to describe the contact resistance at the nanoparticle-electrode junction, and charge transfer resistance that used to describe electron/ion transport within the nanoparticle. We had performed further control experiments to demonstrate this point. For example, the enhanced electrical contact (via vacuum drying), or the change in the offset potential, was able to monitor R_{NP} as expected.

12. The equivalent circuit model of single PBNPs

It can be seen from the equivalent circuit (Fig. 4b) that impedance can be written as:

$$Z_F = R_{NP} + \frac{1}{j\omega C_{NP}}.$$

When a voltage with amplitude of V_0 was applied, the current is:

$$I_F = \frac{V_0}{Z_F} = \frac{V_0}{R_{NP}-j\left(\frac{1}{\omega C_{NP}}\right)} = \frac{V_0\omega C_{NP}}{R_{NP}\omega C_{NP}-j} = \frac{V_0\omega C_{NP}(R_{NP}\omega C_{NP}+j)}{(R_{NP}\omega C_{NP})^2+1}.$$

The real and imaginary parts of the current can be written as:

$$I_{F,Re} = \frac{V_0\omega^2 {C_{NP}}^2 R_{NP}}{(R_{NP}\omega C_{NP})^2+1}, \quad I_{F,Im} = \frac{V_0\omega C_{NP}}{(R_{NP}\omega C_{NP})^2+1}.$$

The amplitude and phase of the current are:

$$|I_F| = \sqrt{{I_{F,Re}}^2 + {I_{F,Im}}^2} = \sqrt{\frac{(V_0\omega C_{NP})^2((R_{NP}\omega C_{NP})^2+1)}{((R_{NP}\omega C_{NP})^2+1)^2}} = \frac{V_0\omega C_{NP}}{((R_{NP}\omega C_{NP})^2+1)^{1/2}}.$$

$$tan\,(\Phi_{I_F}) = \frac{I_{F,Im}}{I_{F,Re}} = \frac{1}{\omega C_{NP}R_{NP}}.$$

According to the current, the amplitude and phase of the charge can be obtained:

$$|Q_F| = \left|\frac{I_F}{2\pi f}\right|, \quad \Phi_{Q_F} = -\frac{\pi}{2} + \Phi_{I_F}.$$

Finally:

$$|Q_F| = \frac{V_0 C_{NP}}{(1+(2\pi C_{NP}R_{NP}\cdot f)^2)^{1/2}}, \qquad \Phi_{Q_F} = tan^{-1}\left(\frac{1}{2\pi C_{NP}R_{NP}\cdot f}\right) - \frac{\pi}{2}.$$

13. Scattering intensity and equivalent radius of different PBNPs

The length and width of PBNPs can be obtained from the scanning electron microscope, and the equivalent radius is calculated by the following formula to assess the volume of PBNP:

$$r = \sqrt{S/\pi},$$

which is considered as the radius of a virtual circle with the same projection area of the nanoparticle. In the 12 nanoparticles which the length was ranging from 100 nm–300 nm, an obvious linear relationship between the scattering intensity and r^3 was observed (Supplementary Fig. 20h). This revealed that the volume of nanoparticles (r^3) was proportional to the scattering intensity. So we could use the scattering intensity instead of the actual volume of particles for further statistics because the AFM imaging took a long time to capture.

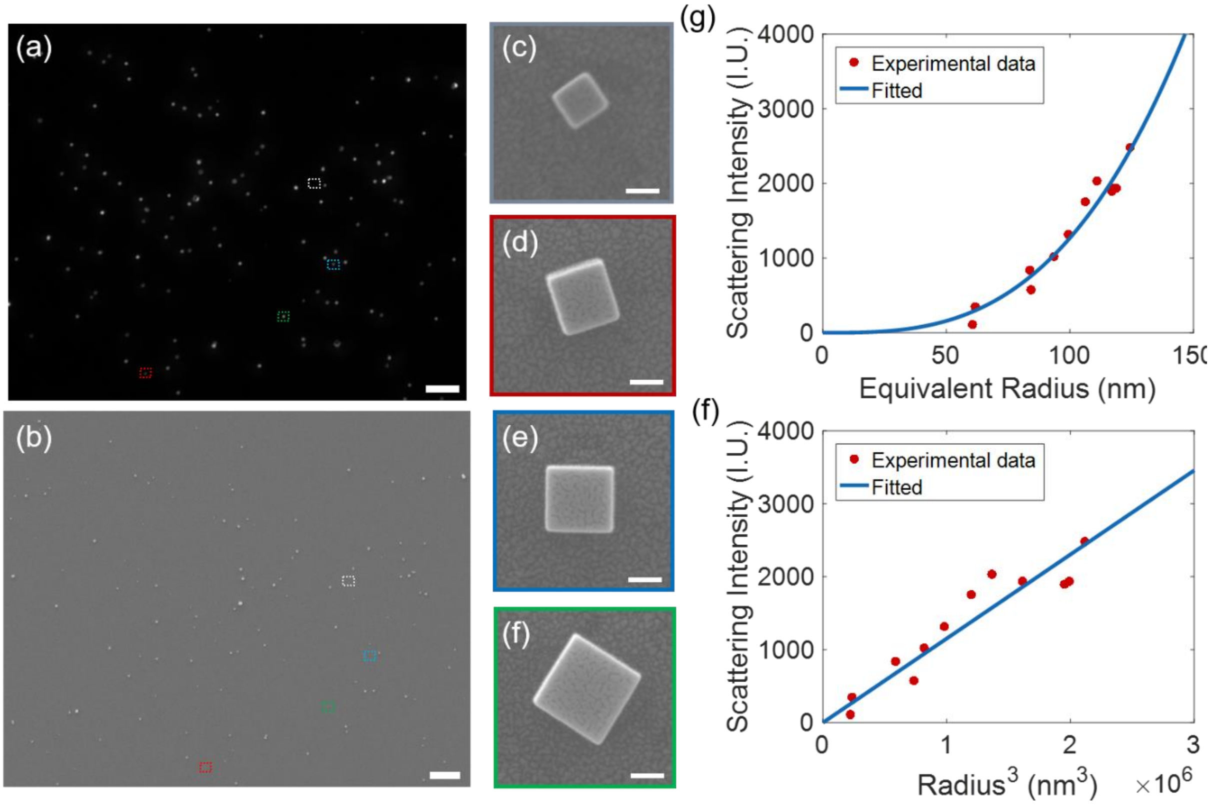

Supplementary Fig. 20 (a) Scattering intensity image of corresponding imaging area, scale bar: 10 μm; (b) SEM image of dark field imaging area, scale bar: 10 μm; (c) - (f) SEM image of single PBNP in (a), indicating the grey, red, blue and green area respectively, scale bar: 100 nm. The scattering intensity of individual PBNPs verse (g) equivalent radius *r* and (h) r^3 estimated from SEM results. The blue line shows the fitted results, and the red dots are experimental data.

14. The calculation of optical coefficient a

Assuming that PBNP can completely convert to PWNP when a sufficiently negative potential was applied, we can see that the scattering intensity of the particle was reduced by 2095 IU in Fig. 1c. Since the following reaction occurred in the one unit cell of PBNP:

$$\mathrm{K_4Fe_4^{III}\left[Fe_4^{II}(CN)_{24}\right] + 4e^- + 4K^+ = K_8Fe_4^{II}\left[Fe_4^{II}(CN)_{24}\right]},$$

this means that one unit cell accepts 4 electron and ions in the reduction process. The total number of cells in PBNP are $(\frac{215}{1.02}) \times (\frac{215}{1.02}) \times (\frac{200}{1.02})$, so the total amount of transferred charge is $4 \times (\frac{215}{1.02}) \times (\frac{215}{1.02}) \times (\frac{200}{1.02})$. By virtue of these above, we can establish an optical electrochemical signal conversion model to obtain the photoelectric conversion coefficient a: 6.0×10^{-5} IU/charge.

15. The equivalent circuit model of the whole entire ITO electrode

We measured the impedance of the whole electrochemical cell (two identical ITO electrodes) with the voltage amplitude of 10 mV, ranging from 0.01 Hz to 10 kHz. A 0.5 M KNO_3 solution was used as electrolyte throughout the work in the absence of pH buffer. Besides, we clarified that the electrochemical measurements were performed in the absence of additional redox probe in the solution.

The equivalent circuit diagram (Fig. 4a) is composed of electric double layer capacitance C_{dl}, electrolyte solution resistance R_s, polarization resistance R_p, and Warburg impendence Z_w. The total impedance is:

$$Z = R_s + \frac{1}{1/(R_p + Z_W) + j\omega C_{dl}}, \quad Z_W = \frac{1}{(j\omega)^n R_W}.$$

Here, n is an adjustable constant and R_W is a constant. When n is 0.5 or 1, this indicates ideal Warburg impendence or pure capacitive behavior respectively. From the simulation results, we can find there is no significant voltage drop in the frequency ranging from 0.01 Hz to 100 Hz (Fig. 4c).

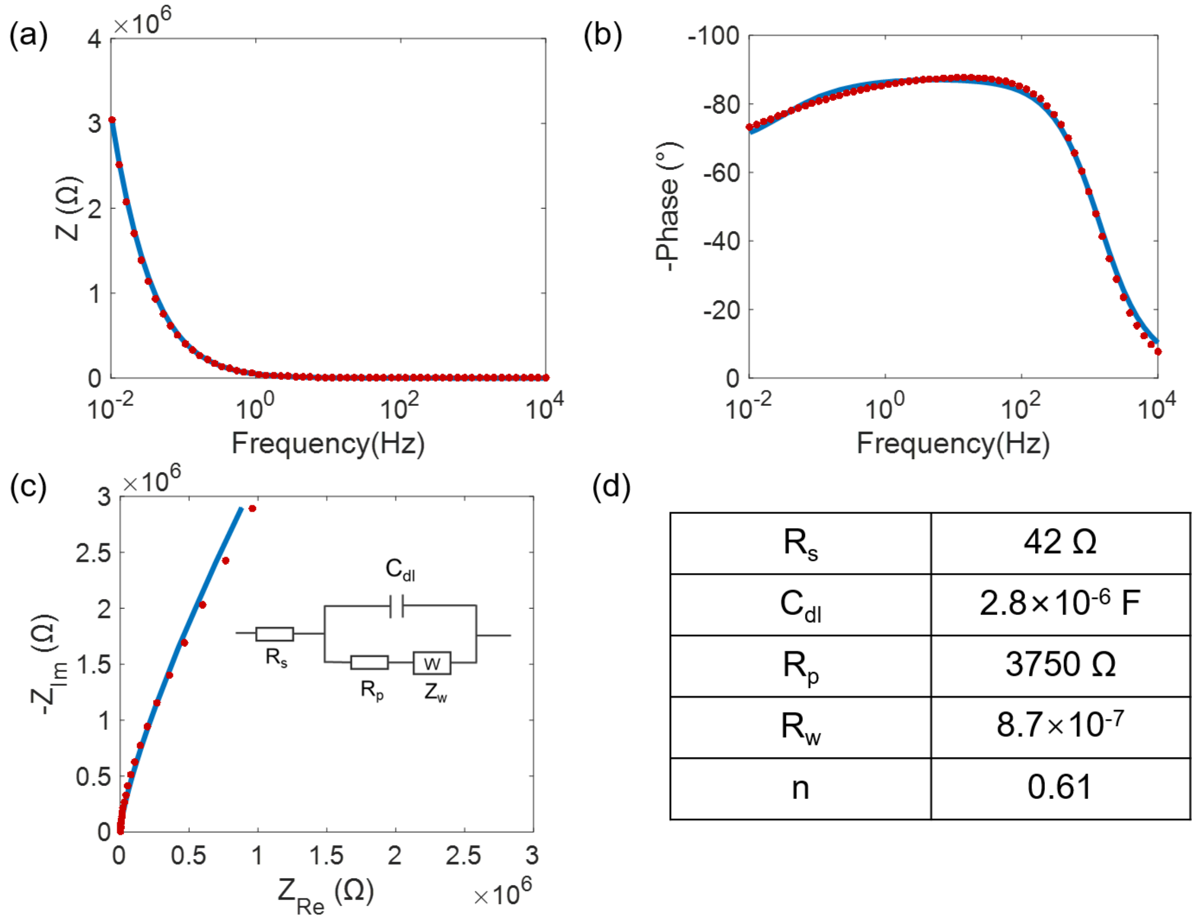

R_s	42 Ω
C_{dl}	2.8×10^{-6} F
R_p	3750 Ω
R_w	8.7×10^{-7}
n	0.61

Supplementary Fig. 21 Bode plots of impedance amplitude (a) and phase (b) of the overall electrochemical cell. (c) The corresponding Nyquist plot, the left is equivalent circuit. (d) Parameter values of the equivalent circuit. The blue line shows the fitted results, and the red dots are experimental data.

Resistor (R_S) described the solution resistance, which was determined to be ~42 Ohm. This value was consistent with literatures that used the same electrolyte and electrochemical cell design[2]. Double layer capacitor (C_{dl}) described the effect of electrical double layer, which was determined to be 2.8 μF. The capacitance density was therefore 4.7 μF/cm^2. Polarization resistor (R_p) in series of warburg element (Z_w) were frequently used to describe the interfacial Faradaic reaction involving the reduction of dissolved oxygen.

It was found that, the reduction of dissolved oxygen was responsible for the deviation of -90° phase at low frequency range as shown in Supplementary Fig. 21b. As long as the oxygen was removed from the solution by purging Argon bubbles, the deviation was reduced (Supplementary Fig. 22).

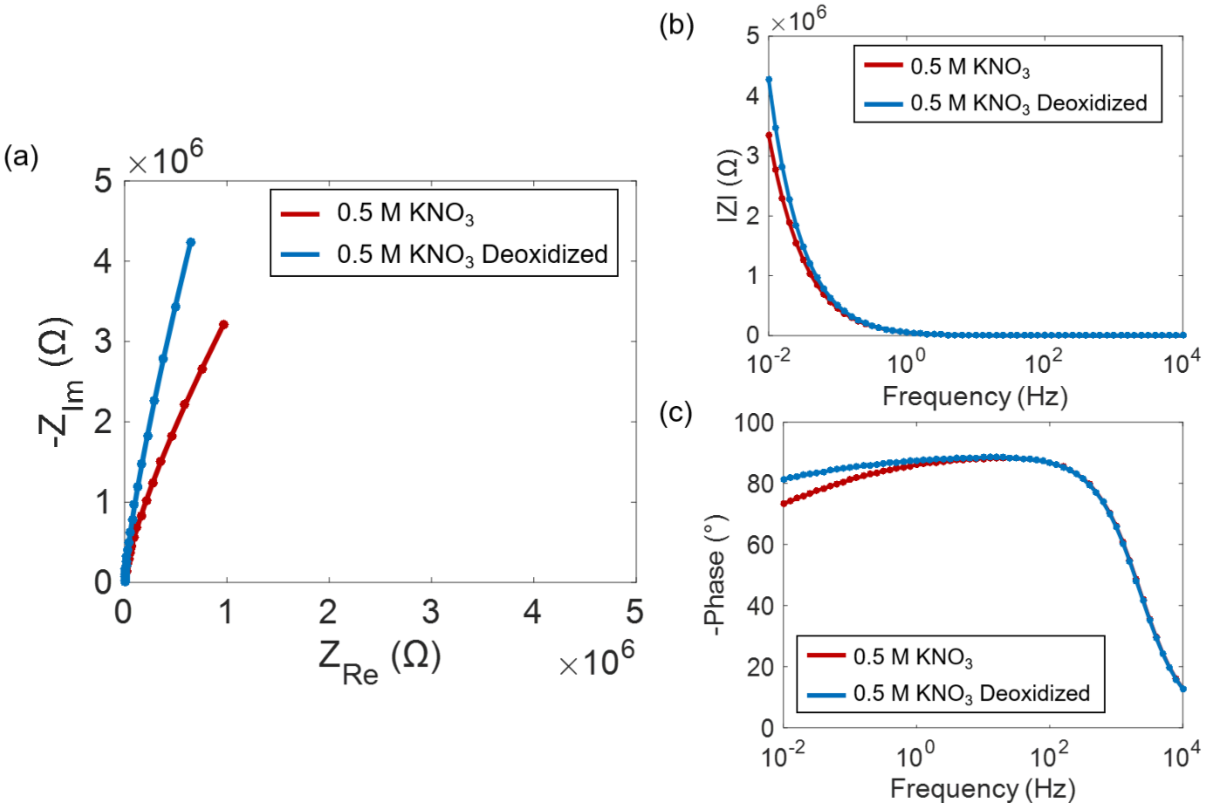

Supplementary Fig. 22 (a) The Nyquist plots of the overall electrochemical cell impedance before (red) and after (blue) removing the dissolved oxygen. The corresponding Bode plots of amplitude (b) and phase (c) are also provided.

Supplementary References

1 Wu, X.; Cao, M.; Hu, C.; He, X., Sonochemical Synthesis of Prussian Blue Nanocubes from a Single-Source Precursor. *Cryst. Growth Des.* **6,** 26-28 (2006).

2 Liu, T.; Li, M.; Wang, Y.; Fang, Y.; Wang, W., Electrochemical impedance spectroscopy of single Au nanorods. *Chem. Sci.* **9***,* 4424-4429 (2018).

3 Jiang, W.; Wei, W.; Yuan, T.; Liu, S.; Niu, B.; Wang, H.; Wang, W., Tracking the optical mass centroid of single electroactive nanoparticles reveals the electrochemically inactive zone. *Chem. Sci.* **12**, 8556-8562 (2021).

4 Sun, L., et al., Collision and Oxidation of Single $LiCoO_2$ Nanoparticles Studied by Correlated Optical Imaging and Electrochemical Recording. *Anal. Chem.* **89**, 6050-6055 (2017).

5 Anderson, T.J. & Zhang, B., Single-Nanoparticle Electrochemistry through Immobilization and Collision. *Acc. Chem. Res.* **49**, 2625-2631 (2016).

6 Merryweather, A.J., Schnedermann, C., Jacquet, Q., Grey, C.P. & Rao, A. Operando optical tracking of single-particle ion dynamics in batteries. *Nature* **594**, 522-528 (2021).

7 Wei, W. et al. Accessing the Electrochemical Activity of Single Nanoparticles by Eliminating the Heterogeneous Electrical Contacts. *J. Am. Chem. Soc.* **142**, 14307-14313 (2020).
